# Food hygiene research: a bibliometric comparison in Iranian and international “Environmental Health” journals

**DOI:** 10.1186/s41043-022-00339-1

**Published:** 2023-01-06

**Authors:** Zahra Aghalari, Hans-Uwe Dahms, Mika Sillanpää

**Affiliations:** 1 Faculty of Public Health, Babol University of Medical Sciences, Babol, Iran; 2 Department of Biomedical Science and Environment Biology, College of Life Science, Kaohsiung Medical University, Kaohsiung, 80708 Taiwan; 3 Research Center for Precision Environmental Medicine, KMU - Kaohsiung Medical University, Kaohsiung, 80708 Taiwan; 4 Department of Marine Biotechnology and Resources, National Sun-Yat-Sen University, Kaohsiung, 80424 Taiwan; 5 Department of Biological and Chemical Engineering, Aarhus University, Nørrebrogade 44, 8000 Aarhus C, Denmark; 6 Faculty of Science and Technology, School of Applied Physics, University Kebangsaan Malaysia, 43600 Bangi, Selangor Malaysia; 7 School of Chemistry, Shoolini University, Solan, Himachal Pradesh 173229 India

**Keywords:** Food hygiene, Community health, Environmental health, Journals

## Abstract

**Background:**

Food hygiene is one of the specialized fields of environmental health, and despite the problems associated with foodborne illnesses, there is no evaluation available that would focus on specialized environmental health journals. The purpose of the present survey is a comparison of the status of food hygiene articles published in Iranian and international journals of environmental health.

**Methods:**

This cross-sectional descriptive study was performed on all published articles in five Iranian environmental health journals and three international environmental health journals that are among the top 5% and 10% based on SNIP, emphasizing the issue of food. Our data were collected by searching relevant keywords in the articles published during the years (2008–2021), with emphasis on food hygiene. In the checklist, journal and articles information was collected by year of publication, a number of articles, information on authors' participation status in terms of number, gender, organizational affiliation, country and continents, and research centers according to authors' authorship. Statistical analysis of data was performed using descriptive and inferential statistical indices. VOSviewer software was also used to visualize the data.

**Results:**

In Iranian environmental health journals, out of 2305 articles (7.3%) and out of 6898 articles in international environmental health journals (2.4%) dealt with food hygiene. Food hygiene articles were divided into seven categories, with the largest number of articles on aquatic and agricultural products each with a frequency of 48 articles. Articles related to heavy metals in food were provided by 30.81%. In this study, out of 150 articles, 15 articles were written with the participation of 30 authors from seven continents (Asia, America, and Europe), most of which were from Asia and India. In international environmental health journals, among the main research topics in articles related to food hygiene, the highest number (52.5%) was related to a determination about pollution such as heavy metal concentrations in food.

**Conclusions:**

Articles published in Iranian and international environmental health journals about food hygiene were limited. According to the increasing prevalence of foodborne illnesses, especially in recent decades, and the importance of paying attention to food hygiene, more targeted studies are needed.

## Introduction

Attention to food hygiene and safety is an important principle for the prevention of food contamination which are caused by diverse microorganisms belonging to bacteria, viruses, parasites, and fungi [1, 2]. The world health organization views foodborne illnesses as one of the most important health problems in the contemporary world [3], and the slogan for environmental health by the information of the Federation of Environmental Health in 2018 was global food safety and sustainability [4, 5]. The prevalence of food contamination in developing and developed countries is between 6 and 73%, respectively [6].

Foodborne illnesses indicate widespread public health problems in both developed and developing countries, but these problems have a greater impact on health and the economy in developing countries [7, 8]. Since Iran is one of the developing countries, it undoubtedly imposes problems on the health system as well as the economy of the country due to foodborne diseases. Therefore, it is necessary to identify and eliminate factors affecting food contamination and unhealthy foodborne diseases with regard to the nutrition of Iranian people.

One of the ways to do targeted research to identify and solve food insecurity and health problems is to study scientific products [9]. Scientometrics or systematic content analysis of scientific documents is one of the new scientific approaches used to monitor scientific activities and manage research in different fields of science [10]. In fact, one of the standard tools for measuring and evaluating scientific output in different fields of science is the use of scientometric indices. Quantitative and qualitative investigations of scientific products in various scientific fields can be done by applying scientific approaches [11]. Scientometrics involves identifying scientific topics and subdivisions in different disciplines, identifying emerging fields of research, examining trends in the development of disciplines over time, or geographical and organizational distribution of scientific products [12]. Studies of the content analysis of articles related to foodstuffs are provided by Guerrero-Bote et al. who studied articles published in the field of food science in the SCImago journal and country rank from 2003–2013 [13] and the study of Vellaichamy et al. about scientometric analysis of food and nutrition research from 1982 to 2012 on articles published in SCOPUS [14].

As the subject of food hygiene is one of the specialized disciplines of environmental health, and despite problems with foodborne diseases, no study has been found in Iran in this regard. The purpose of the present study was to compare the status of food hygiene articles published in Iranian and international journals of Environmental Health.

## Methods

### Research design

This scientometric cross-sectional study was conducted retrospectively over a fourteen years period (2008–2021) on articles published in specialized environmental health journals. The inclusion criteria for this study were scientific journal research, environmental health in the title of the journal, having at least four issues per year and publication of at least three consecutive years. According to these criteria, three Persian-language journals, the Iranian Journal of Health and Environment (IJHE), the Journal of Environmental Health Engineering (JEHE), the Journal of Research in Environmental Health (JREH), and the two English-language journals, Environmental Health Engineering and Management Journal (EHEMJ) and the Journal of Environmental Health Science and Engineering (JEHSE) were reviewed.

In order to compare articles among Iranian journals with the world's top environmental health journals, three international journals, which are among the top 5% and 10% based on SNIP in environmental journals, were examined. The top journals reviewed here include Environmental Health Perspectives Journal (EHP) published by the USA with an impact factor 8.05 in 2018 and with H-Index 249, Journal of Toxicology and Environmental Health—Part B: Critical Reviews (JTEH) with an impact factor of 6.436 in 2018, published by the UK with an H-Index of 70, and Environmental Health Journal (EH) with impact factor 4.430 in 2018, published by the USA with an H-Index of 73.

### Questionnaire design and data collection

**Fig. 1:**
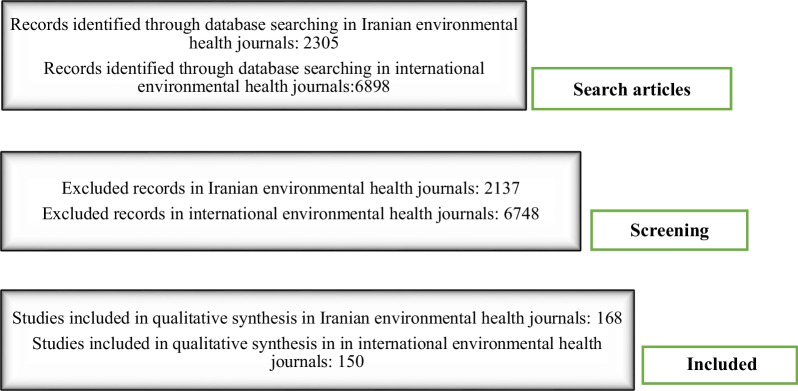
Flowchart of study design

The data were collected by surveying the sites of each journal, from a census 2008 to the latest issue in 2021. In order to collect data, individual articles were downloaded by visiting each journal's website and the abstracts, the keywords and the full text of the articles were reviewed, and finally, the articles on food hygiene were included (Fig. 1).

Data were collected through a researcher-made checklist used in other studies [9, 10]. The checklist lists important and common variables recommended by the Medical Science Authors Association (ICMJE) [15] and the World Association of Medical Editors (WAME) [16]. Variables studied were the number of articles by journals and year of publication, type of food, main research topic in the articles, type and method of studies, national and international authors' collaboration as well as geographical distribution of researchers in the research.

### Data analysis

Information from individual research papers was coded and entered into excel software after entering the checklist. Descriptive statistics such as the tendency to center and dispersion indices were used for statistical data processing. VOSviewer software was also used to visualize the data and to show the geographical distribution of researcher affiliations (status and extent of participation of researchers in different countries and continents with researchers in Iran). This software helps researchers to visualize the co-authoring networks of authors from different countries. For example, this software enables the display of data density in different colors, their clustering or their dispersion in different geographical locations [17].

## Results

**Fig. 2:**
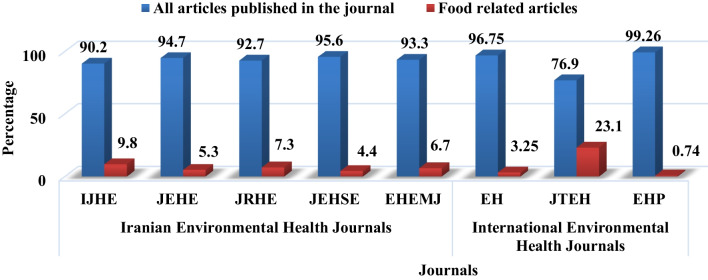
Frequency of articles published on food hygiene in Iranian and international environmental health journals (2008–2021)

In this study, in five environmental science journals, 2305 articles were published in 174 issues over fourteen years. The largest number of articles was in the J Environ Health Sci Engineer (678 articles–42.42% of the total). Of the 2305 articles published in the fourteen years, 168 (7.3%) were devoted to the topic of food hygiene, most of them belonging to IJHE (9.8%) (Fig. 2). Also, 6898 articles were reviewed in three international environmental health journals most of the articles were related to the EHP journal with a frequency of 73.8%. Out of 6898 articles published in international environmental health journals, 168 articles (2.4%) were related to food hygiene, most of them belonging to the JTEH journal (23.1%) (Fig. 2).

**Fig. 3:**
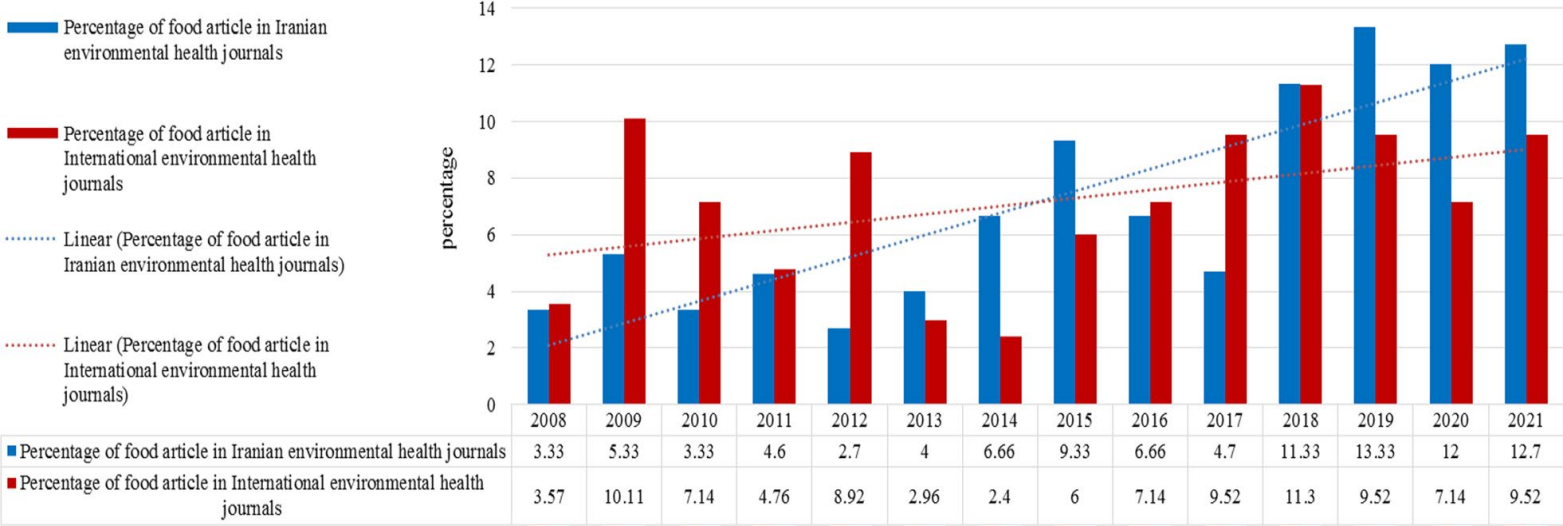
Relative trend of food articles in comparison with all articles published in Iranian and international environmental health journals (2008–2021)

The trend of publishing articles on food hygiene has been fluctuating over the past decade, with most of the publications in Iranian environmental health journals in 2019 (13.33%) and most of the publications in international environmental health journals in 2018 (11.3%) (Fig. 3).

### Type of foods in the articles

In Iranian environmental health journals, five articles that examined authors' knowledge, attitude, and practice about food hygiene were categorized into general food products because of the unclear type of food. The largest number of articles on aquaculture and agricultural products had 48 articles each. Of the main research topics in articles related to food hygiene, the highest number (39.34%) was related to the determination of heavy metal concentrations in food (Table 1). In international environmental health journals, the largest number of articles with 57 articles was about agricultural products. Of the main research topics in articles related to food hygiene, the highest percentage (34.52%) was related to determine the amount of chemicals in food (Table 1).

**Table 1:** Frequency of food types and main research topics in published studies about food hygiene in Iranian and international environmental health journals (2008–2021)

Variables	Level	Iranian environmental health journals		International environmental health journals	
		Number	Percentage	Number	Percentage
Kinds of food	Seafood	48	32	40	23.8
	Agricultural products	48	32	57	33.93
	Dairy products	20	13.33	16	9.52
	Drinks	13	8.67	22	13.1
	Canned food	10	6.66	15	8.92
	Food products	7	4.67	8	4.77
	Livestock products	4	2.67	10	5.96
The main topic of research in the articles	Determination of heavy metal concentration	59	39.34	39	23.2
	Determine the amount of chemicals	46	30.66	58	34.52
	Microbial tests	22	14.66	35	20.83
	Knowledge, attitude, and practice of people about food hygiene	5	3.34	9	5.35
	Microbial testing and determination of heavy metal concentrations	18	12	27	16.1

### Type, method of study, and data collection tools

Content analysis of the articles in Iranian environmental health journals showed that all articles were original research articles. In terms of the method of study, the majority with 138 articles (92%) were empirical and 12 were semiempirical or descriptive (8%). In 138 articles (92%) the data were collected in the laboratory, and in 12 articles (8%) the data were collected by questionnaire and checklist.

In international environmental health journals, all articles were original research. In terms of the method of study, the majority with 149 articles (88.7%) were empirical and semiempirical. In 88.7% of the articles, the data were collected by laboratory-based methods and in 11.3% of articles, the data were collected by questionnaire and checklist.

### National and international author’s collaboration in Iranian environmental health journals

In writing 150 articles on food hygiene, 473 authors collaborated, 317 (67.1%) were male and 156 (32.9%) were female. The minimum and the maximum number of male authors per article were 1 to 9 (2.77 ± 2.25). The minimum and the maximum number of female authors were 0 to 4 authors (1.04 ± 1.03).

Of the 473 authors, 191 were cited in 55 articles from research centers. A total of 19 research centers were listed in the articles, of which only five were called food Quality Research Centers. There were also two research centers affiliated with the University of India (Table 2).

**Table 2:** Frequency of authors participation in research centers on food hygiene articles in Iranian journals for environmental health

Country	University	Research centers	Number of researchers with efficacy center
Iran	Tehran University of Medical Sciences	Environmental Research Center (Water, Air, Solid Waste Research Centers)	29
		Food Quality Research Center	16
	Kermanshah University of Medical Sciences	Environmental Factors Affecting Health	7
		Social Development and Health Promotion	3
	Isfahan Medical Sciences	Food Quality Research Center	5
	Rasht University	International Aquatic Research Institute	8
	Shiraz University of Medical Sciences	Environmental Health Research Center	2
	Kerman University of Medical Sciences	Environmental Health Research Center	3
	Kurdistan University of Medical Sciences	Cellular and Molecular Research Center	2
	Mazandaran University of Medical Sciences	Pharmaceutical Research Center	5
	Shiraz University	Agricultural Education Research Center	1
	Zanjan University	Environmental Research Center	3
Non-Iranian	India University	Ecological Studies	1

In terms of the number of Iranian authors contributing to other countries on food hygiene, of the 150 articles written by 473 researchers, 15 articles were written by 30 authors from 7 Asian, USA, and European countries, most with eight articles by Asian researchers and 11 authors (excluding Iranian authors). Indian researchers (with seven articles and nine authors) have had most research collaborations with Iran.

**Fig. 4:**
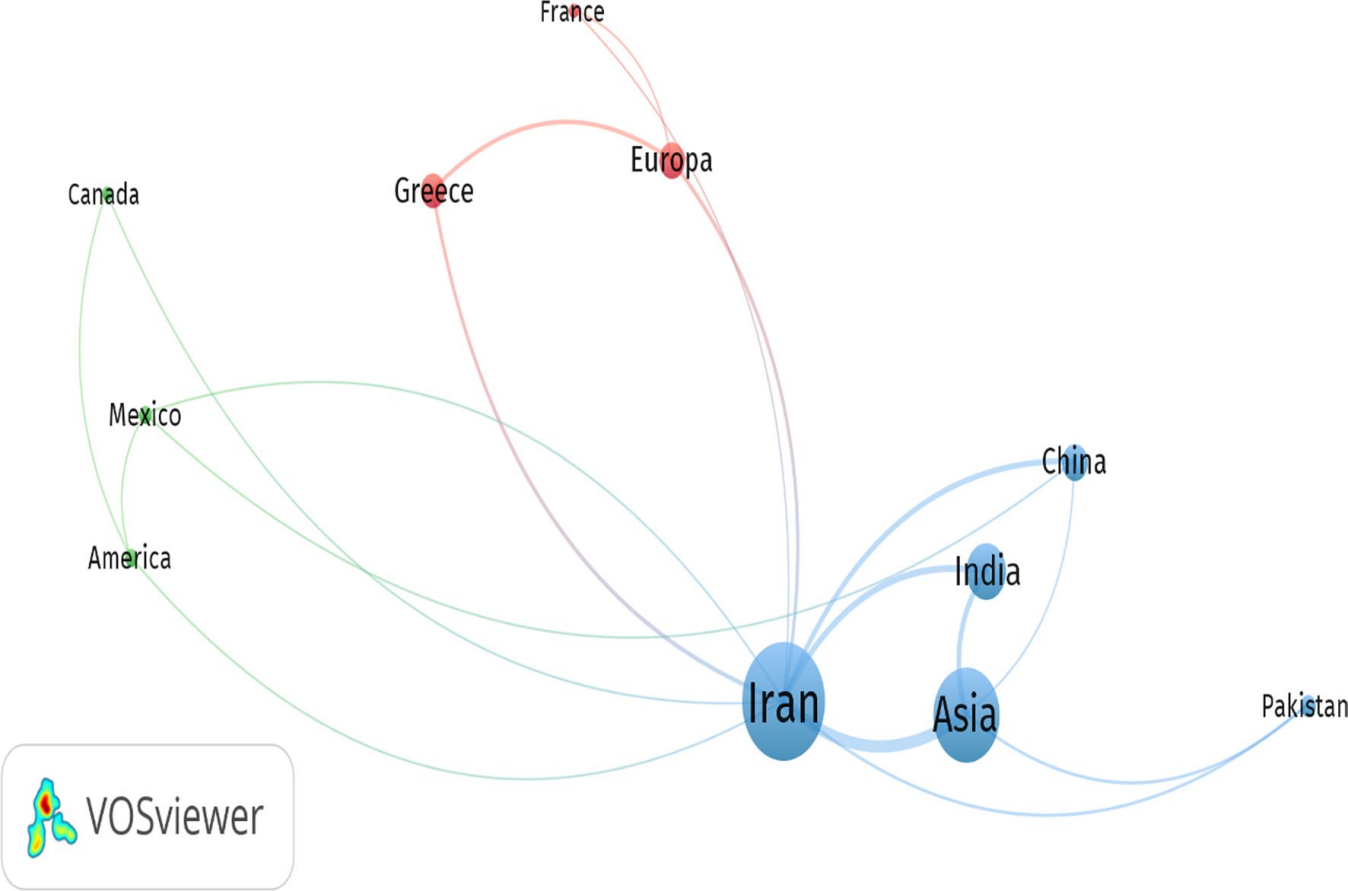
Network of authors collaborating across countries and continents on food hygiene articles in Iranian journals of environmental health (2008–2021)

Data analysis with VOSviewer software showed the number of articles from each country and continent shared with Iranian researchers, and the diameter of the link between the two nodes is proportional to the number of co-authored articles. When cooperation of countries with Iran was considered, Iran was placed at the center of the nodes with 15 articles. The map showed that countries with more participation of Iranian authors were closer to Iran, and countries with less participation were shown in smaller fonts and further away (Fig. 4).

## Discussion

The findings showed that in Iranian environmental health journals, out of 2305 articles (7.3%) and out of 6898 articles in international environmental health journals (2.4%) were focused on food hygiene. In a study by Overbey et al. on the use of social media for food hygiene education, the findings showed that over a 5-year period, out of 184 articles, 24 (13.04%) were related to food hygiene [18]. In a study by Lytle et al. comparing food hygiene in PubMed, EMBASE, PsycINFO, and global health databases over a nine-year period, the authors showed that out of 11,928 articles on food hygiene, 432 articles (3.62%) were about food hygiene [19]. A comparison of the findings of these studies with the findings of the present approach indicates the deficiencies of food hygiene research and calls for the attention of nutritionists and food safety specialists.

The trend of article publications on food hygiene has been fluctuating, but overall, the publication of food articles in the journals under review has increased from 3.33 to 12.7% within the 14 years of the survey. The study of Dabirian et al. showed an upward trend in the publication of food articles in the journal of food product marketing. According to that study, publication records increased from 17 to 32 percent over the past 23 years [20]. The reasons for the recent rising trend in food articles are lifestyle changes of people, the use of low-quality foods such as fast food, and the subsequent rise of nutrition-related illnesses, which has attracted the attention of nutritionists and researchers.

In Iranian environmental health journals, 14.66% of articles and in international environmental health journals 20.83% focused on microbial testing and bacterial contamination of food. A study by Sweileh et al. on the SCOPUS database stated that of 5522 articles found on *Campylobacter*, 4.46% of articles on food contamination with this bacterium were published within the last 15 years [21]. Torgerson et al. reported within the WHO's estimate of foodborne parasitic diseases that 48% of the articles and reports found on foodborne parasitic diseases were published between 2010 and 2015 [22]. A study by Osei-Tutu et al. reported that in Colombia over 5 years, the prevalence of food-related diseases ranged from 2.56 to 11.5% [23]. Diseases and toxicities from eating unhealthy foods were increasing within recent years. Recent censuses in the UK show that food poisoning caused by the bacteria, *Salmonella*, *Campylobacter*, and *Listeria*, were increasing. As a result of this increasing trend, the British government has decided to launch a project called food safety through the Ministry of Health [24]. Therefore, it is recommended that environmental health experts monitor food quality regularly, especially in food preparation and distribution areas such as restaurants.

According to the findings of this study, in Iranian environmental health journals out of 473 authors, 67.1% were male and 32.9% were female. In a study by West et al., the authors of scientific articles were reported to be 21.9% female authors [25]. Amrein's research on top 60 medical journals in the Reuters database showed that women were on the editorial board of medical journals, with only 15.9% female editors and less than 20% of the 60 female journals [26]. In line with the results of these studies, the results indicate the substantial contribution of Iranian female researchers in conducting research, including health research in Iran.

In Iranian environmental health journals, out of 150 articles, 15 articles were written with the participation of 30 authors from seven continents (Asia, America and Europe), most of which were from Asia and India. In a study by Tirgar et al., the status of international collaborations by Iranian researchers in compiling environmental health engineering articles with Asian researchers was 63.5% higher than that of other continents [9]. The reason for the extensive collaboration of Iranian researchers with Asian researchers can be attributed to the similarities of environmental problems, the education of many Iranian experts in Asian countries, favorable socio-political relations, and close proximity to Asian countries. A study by Royle et al. indicated that one of the reasons for the researchers' involvement in writing scientific papers was geographical proximity of countries [27].

One of the strengths of this study is the use of scientometric and citation analysis methods to evaluate the status of scientific work on food hygiene that has been less discussed in environmental health issues. The strengths of this study were also the analysis of a relatively wide range of time (for fourteen years), the review of articles in all specialized environmental health publications, and the use of advanced VOSviewer software. One of the limitations of this study is the lack of a review of articles on food hygiene in specialized nutrition-related journals, which can be explained by the limited number of specialized journals.

## Conclusions

Articles published in Iranian and international environmental health journals about food hygiene are limited. According to the increasing prevalence of foodborne illnesses, especially in recent decades, and the importance of paying attention to food hygiene, more targeted studies are needed. Therefore, interdisciplinary collaboration between environmental health professionals and nutritionists and interagency collaboration between the food and drug administration and the universities of medical sciences and health departments food health with more emphasis are suggested.

### Limitations

Since this systematic review was focusing on food hygiene articles that are present in “Environmental Health” journals, it witnesses a bias of topics toward, e.g., medicine, community and public health and infectious diseases. This way aspects of policy and governance, cost-effectiveness, and meta-analysis were exempted by and large.

## Data Availability

The datasets used and analyzed during the present study are available from the corresponding author upon reasonable request.

## Abbreviations

EHEMJ: Environmental Health Engineering and Management Journal
IJHE: Iranian Journal of Health and Environment
JEHE: Journal of Environmental Health Engineering
JEHSE: Journal of Environmental Health Science and Engineering
JREH: Journal of Research in Environmental Health
ICMJE: International Committee of Medical Journal Editors
WAME: World Association of Medical Editors
